# Supplementary material for: New In Vitro Methodology for Kinetics Distribution Prediction in the Brain. An Additional Step towards an Animal-Free Approach
Source: Animals (Basel). 2021 Dec 10;11(12):3521. doi: 10.3390/ani11123521 (PMC8697921; doi:10.3390/ani11123521)
Supplement: Supplementary file 1 [file animals-11-03521-s001.zip › animals-1481354-supplementary.pdf]

Table S1. Chromatographic conditions validation.

	r^2	LLQ (μM)	Accuracy	Precision	REF
Amitriptyline	0.996	8.20	6.1	3.2	[1]
Atenolol	0.996	1.49	6.3	5.1	
Carbamazepine	0.994	0.76	3.9	3.6	[1]
Fleroxacin	0.997	0.05	6.0	5.2	[1]
Loperamide	0.995	2.65	4.0	4.5	
Norfloxacin	0.991	2.42	3.9	4.9	
Pefloxacin	0.998	0.61	3.9	3.7	[1]
Propranolol	0.999	5.74	3.9	3.4	
Zolpidem	0.997	4.30	6.3	4.8	[1]

1. Sánchez-Dengra, B.; Gonzalez-Alvarez, I.; Bermejo, M.; Gonzalez-Alvarez, M. Physiologically Based Pharmacokinetic (PBPK) Modeling for Predicting Brain Levels of Drug in Rat. *Pharmaceutics* **2021**, *13*, 1402, doi:10.3390/pharmaceutics13091402.
